# A rare lung disease diagnosed by CT in a young pregnant woman

**DOI:** 10.1002/ccr3.5071

**Published:** 2021-11-10

**Authors:** Sunil Persad, Nadiya Dowlath, Michael Prasad, Vanessa Harry

**Affiliations:** ^1^ Faculty of Medical Sciences University of West Indies St Augustine Trinidad; ^2^ Department of Obstetrics and Gynecology San Fernando General Hospital San Fernando Trinidad

**Keywords:** high‐resolution CT, LAM, lymphangioleiomyomatosis, pneumothorax

## Abstract

Lymphangioleiomyomatosis (LAM) is an unusual lung disease which can be diagnosed by its characteristic appearance on a high‐resolution CT, and may not always require a biopsy.

## Graphical abstract

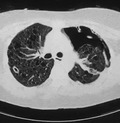

## 1 QUESTIONS AND TEXT

Lymphangioleiomyomatosis (LAM) is a rare cystic lung disease that predominantly affects women in the reproductive age group, many of whom may present with a pneumothorax^1^. High‐resolution CT is known to provide a correct diagnosis in over 80% of patients.

**FIGURE 1:**
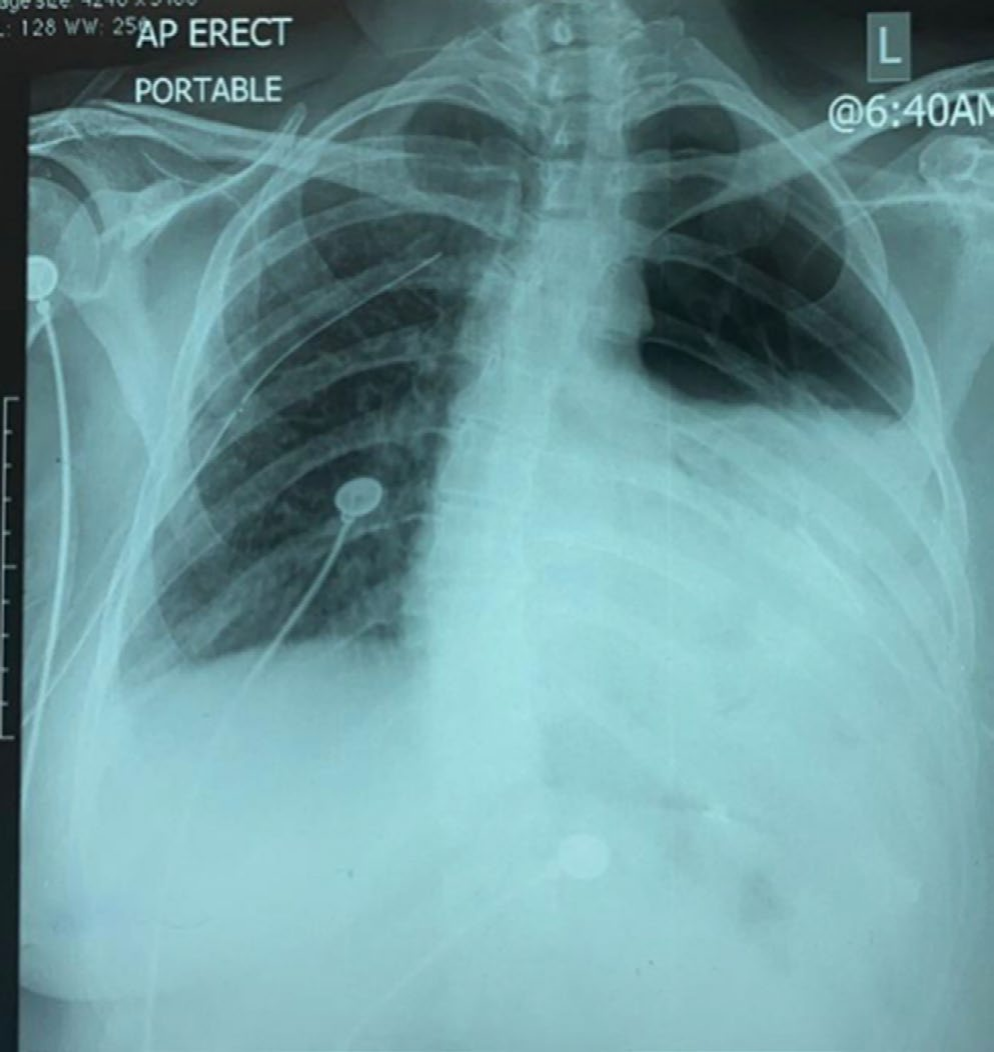
Chest X‐ray showing left pneumothorax

**FIGURE 2:**
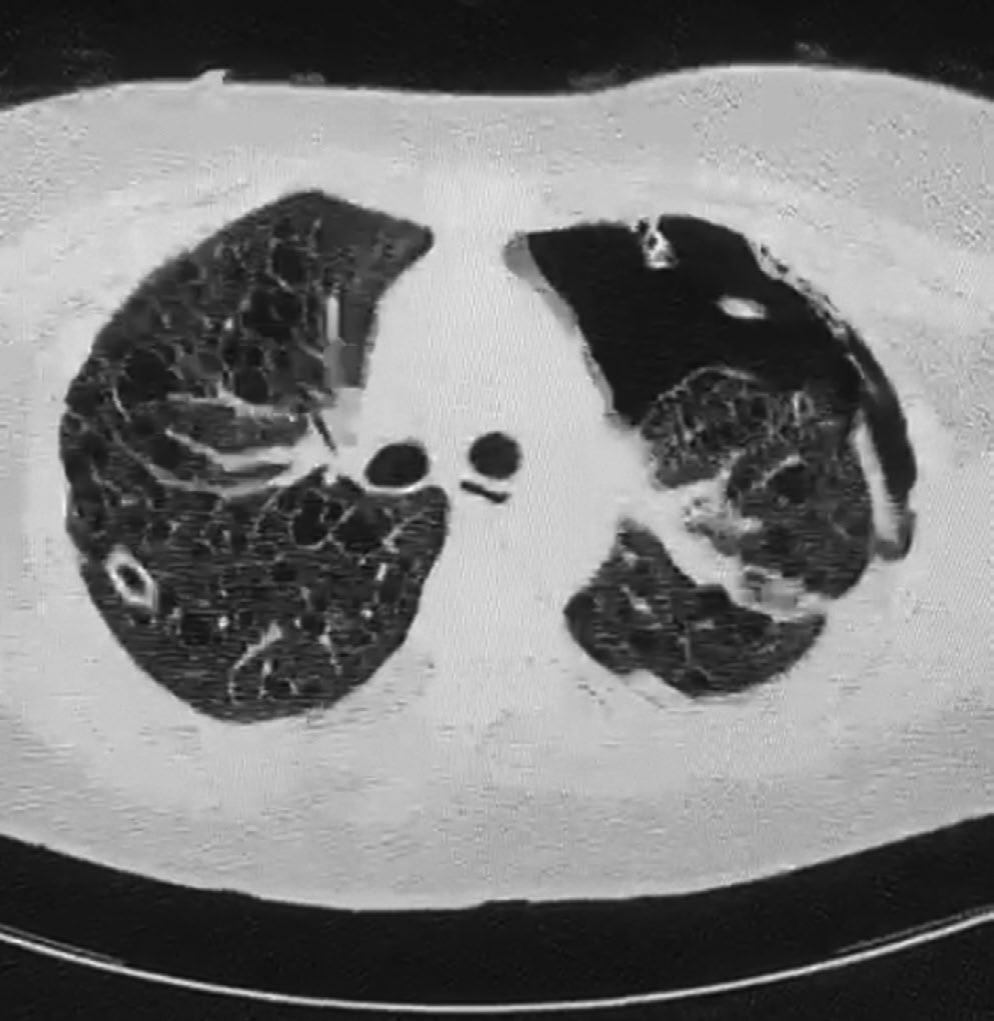
CT scan showing characteristic appearance of LAM

Q1. What is the diagnosis based on the Chest X‐ray shown in Figure 1?
Q2. What is the diagnosis based on the high‐resolution CT shown in Figure 2?

A 38‐year‐old woman in her second pregnancy at 20 weeks gestation presented to our maternity unit with a history of shortness of breath and chest tightness.^1^ Physical examination showed decreased air entry on the left side of the chest and her oxygen saturation was 90% on room air.

A chest X‐ray (Figure 1) revealed a left‐sided pneumothorax, and she went on to have a CT scan of the chest (Figure 2). This showed multiple thin‐walled cystic lesions in both lung fields with a large left‐sided pneumothorax as well as a chylothorax.

These appearances are consistent with a diagnosis of lymphangioleiomyomatosis, a rare multisystem disease that can occur either sporadically or in association with tuberous sclerosis complex.

In 2016, the American Thoracic Society and the Japanese Respiratory Society published clinical practice guidelines that state a definitive diagnosis of LAM can be established if the patient has a characteristic high‐resolution CT of the chest.^2^

Therefore, LAM is one of the few diseases in which a diagnosis can be made on solely on imaging, even in the absence of a confirmatory histopathological lung biopsy.

## CONFLICT OF INTEREST

None declared.

## AUTHOR CONTRIBUTIONS

SP, ND, and MP were involved in this patient's clinical management, and they collected the necessary information and drafted the manuscript with VH. VH revised the manuscript, and the final version was approved by all authors.

## ETHICAL APPROVAL

The authors have no ethical conflicts to disclose.

## CONSENT

Informed consent was obtained from the patient for the publication of this clinical image.

## Data Availability

Data freely available.
